# Supplementary material for: Modulation of Equid Herpesvirus-1 Replication Dynamics In Vitro Using CRISPR/Cas9-Assisted Genome Editing
Source: Viruses. 2024 Mar 6;16(3):409. doi: 10.3390/v16030409 (PMC10975850; doi:10.3390/v16030409)
Supplement: Supplementary file 1 [file viruses-16-00409-s001.zip › viruses-2884407-supplementary.pdf]

Table S1: Primer sequences used to amplify EHV-1 T953 target genes.

EHV-1 genes (Acc. No: KM593996)	Primers	Sequence (5´ to 3´)	Product Size (bp)
ORF30 DNA polymerase	Forward	CATAGCACACGTCTGGCAGA	3919
	Reverse	GTTTAAGCAAGGTTGGCGGG	
ORF31 Major DNA-binding protein	Forward	GCCAACCTTGCTTAAACGCT	3823
	Reverse	TACAAACACAACGGGGGAGG	
ORF7 Helicase-primase subunit	Forward	TTGGCAGCAATCCCATCCTT	3600
	Reverse	GTGTTGTCGGCAGAGGAGAT	
ORF74 Glycoprotein E	Forward	ACAGTTCTCCACTTTGGCGT	1854
	Reverse	GAGCCGGATCTGGGATAACG	

ORF: Open Reading Frame; bp: Base pair

Table S2: Sequences of primers used to amplify the sgRNA-CRISPR/Cas9 targeted regions for gene edition confirmation.

Target	Primer direction	Sequence (5´ to 3´)	Expected size (bp)
**sgRNA ORF30_1**	Forward<br>Reverse	CGTCGAGTTTGTAGCTGGGT<br>CTGCCTGGCCCGACTATAAG	555
**sgRNA ORF30_2**	Forward<br>Reverse	CAGCTCGTACTCGCTGTCAA<br>CGACAACTTTCACCCGGAGA	490
**sgRNA ORF30_3**	Forward<br>Reverse	CAAGCAGTGCCTGGGTTTTC<br>CGCACCTCACCGTCTACTTT	405
**sgRNA ORF30_4**	Forward<br>Reverse	GGCCTGGATAATGCTTGGGT<br>ACCCAGCTACAAACTCGACG	598
**sgRNA ORF31_1**	Forward<br>Reverse	CGGAGGTTTGGCTTTTCTGC<br>GGGTGGAGTCAAACAGCGTA	511
**sgRNA ORF31_2**	Forward<br>Reverse	TACTTTGCCAACCTGGTGCT<br>TCTGAACCATCGGTCCTCCT	523
**sgRNA ORF31_3**	Forward<br>Reverse	GACTGTGACGTGCTGGGTAA<br>CACTGGCTGAAACTGGTTGC	507
**sgRNA ORF31_4**	Forward<br>Reverse	GCCTGGATCGACCGTACAAT<br>TCTGAACCATCGGTCCTCCT	412
**sgRNA ORF7_1**	Forward<br>Reverse	TGTACGGTGGCATCGGAAAA<br>CGGCGGCTATTACACATTGC	524
**sgRNA ORF7_2**	Forward<br>Reverse	TCCATGGCCGCATAGTCATC<br>AGCCGGGTTTGGAGAGATTG	453
**sgRNA ORF7_3**	Forward<br>Reverse	CACGTCGACAACAAACGCTT<br>ACGCATGCGAGTGTACAGAA	548
**sgRNA ORF7_4**	Forward<br>Reverse	GCACAATCTCCCTCTCTCCG<br>TGCTAGTGACGAGCCACAAG	431
**sgRNA ORF74_1**	Forward<br>Reverse	ATTCGTGGCCTTCTAGGTGC<br>GTCAGAGTCCGAAGCTAGGC	527
**sgRNA ORF74_2**	Forward<br>Reverse	TACCTCGCCTTTTTGCTGGT<br>TGTTTCTGACAGGGTTGCGT	528
**sgRNA ORF74_3**	Forward<br>Reverse	ATGAAACACTCCGTCGTGCT<br>ACAAACACATACAGCCCGGT	545
**sgRNA ORF74_4**	Forward<br>Reverse	TGGACGTATACGCTGCTGTC<br>CTGGTATGGTGGGAGCTGTG	450

sgRNA: Single-guide RNA; ORF: Open Reading Frame; bp: Base pair

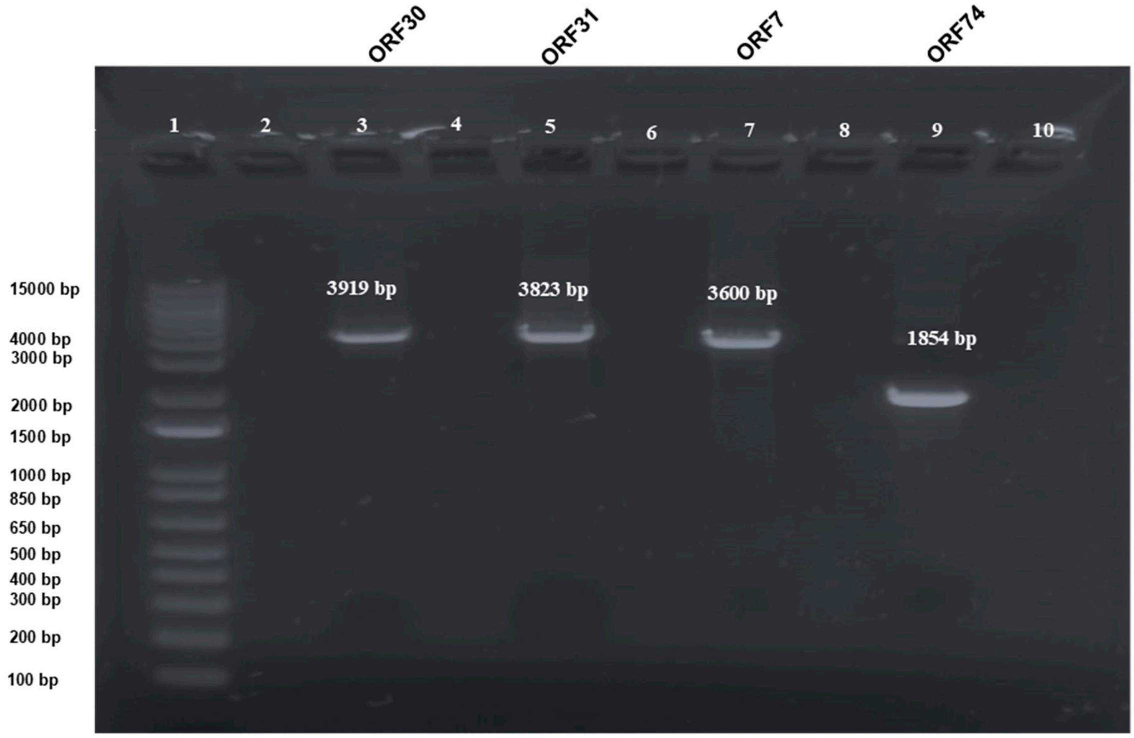

Figure S1: Gel electrophoresis of the EHV-1 target genes (ORF30, ORF31, ORF7 and ORF74) after amplification. Lane (1) 1 Kb plus DNA ladder, lane (3) shows a specific band for ORF30 at 3919 bp, Lane (5) shows a specific band for ORF31 at 3823 bp, Lane (7) shows a specific band for ORF7 at 3600 bp and Lane (9) shows a specific band for ORF74 at 1,854 bp.
